# The Effect of Duration of Lenalidomide Maintenance and Outcomes of Different Salvage Regimens in Patients with Multiple Myeloma (MM)

**DOI:** 10.1038/s41408-021-00548-7

**Published:** 2021-09-22

**Authors:** Matthew Ho, Saurabh Zanwar, Prashant Kapoor, Morie Gertz, Martha Lacy, Angela Dispenzieri, Suzanne Hayman, David Dingli, Francis Baudi, Eli Muchtar, Nelson Leung, Taxiarchis Kourelis, Rahma Warsame, Amie Fonder, Lisa Hwa, Miriam Hobbs, Robert Kyle, S. Vincent Rajkumar, Shaji Kumar

**Affiliations:** 1 Division of General Internal Medicine, Department of Medicine, Mayo Clinic, Rochester, MN USA; 2 Division of Hematology, Department of Internal Medicine, Mayo Clinic, Rochester, MN USA

**Keywords:** Myeloma, Myeloma

## Abstract

The optimal duration of lenalidomide maintenance post-autologous stem cell transplant (ASCT) in Multiple Myeloma (MM), and choice of therapy at relapse post-maintenance, need further evaluation. This retrospective study assessed outcomes of patients with MM (*n* = 213) seen at Mayo Clinic, Rochester between 1/1/2005–12/31/2016 who received lenalidomide maintenance post-ASCT. The median PFS was 4 (95% CI: 3.4, 4.5) years from diagnosis of MM; median OS was not reached (5-year OS: 77%). Excluding patients who stopped lenalidomide maintenance within 3 years due to progression on maintenance, ≥3 years of maintenance had a superior 5-year OS of 100% vs. 85% in <3 years (*p* = 0.011). Median PFS was 7.2 (95% CI: 6, 8.5) years in ≥3 years vs. 4.4 (95% CI: 4.3, 4.5) years in <3 years (p < 0.0001). Lenalidomide refractoriness at first relapse was associated with inferior PFS2 [8.1 (95% CI: 6.4, 9.9) months vs. 19.9 (95% CI: 9.7, 30.2; *p* = 0.002) months in nonrefractory patients]. At first relapse post-maintenance, median PFS2 was superior with daratumumab-based regimens [18.4 (95% CI: 10.9, 25.9) months] versus regimens without daratumumab [8.9 (95% CI: 5.5, 12.3) months; *p* = 0.006]. Daratumumab + immunomodulatory drugs had superior median PFS2 compared to daratumumab + bortezomib [NR vs 1 yr (95% CI: 0.5, 1.5); *p* = 0.004].

## Introduction

The current most widely agreed upon frontline treatment strategy for multiple myeloma (MM) involves induction with triplet therapies followed by high-dose chemotherapy and autologous stem-cell transplantation (ASCT) for eligible patients, and then maintenance therapy with or without preceding consolidation. Lenalidomide is the preferred maintenance strategy following ASCT, especially in non-high-risk patients with MM, and multiple randomized studies (e.g., CALGB100104, IFM2005-02, GEMIMA, Myeloma IX, German multicenter study) have demonstrated an improvement in progression-free survival (PFS) with lenalidomide maintenance [1–5]. Only the CALGB100104 study showed a significant overall survival (OS) improvement with lenalidomide maintenance while an OS benefit was not demonstrated in the other studies; possibly because they were not powered for OS as the primary endpoint [3, 4, 6]. A meta-analysis of CALGB, GEMIMA, and IFM studies confirmed a statistically significant improvement in OS in patients receiving lenalidomide maintenance [7]. While there is no prospective data to guide the optimal duration of lenalidomide maintenance, the current practice consensus is to continue lenalidomide indefinitely until progression or unacceptable toxicity. This is supported by retrospective data from two studies showing improvement in PFS (and OS in one) with a longer duration of lenalidomide maintenance up to 32 months [8, 9]. Importantly, while maintenance therapy has improved outcomes for patients with MM, progression on maintenance therapy is common and there are limited data to guide the selection of optimal therapy at relapse. In this study, we follow-up on patients treated with lenalidomide-based maintenance therapy to assess the impact of duration of maintenance and report the outcomes with various treatment regimens used at first relapse post-maintenance.

## Patients and methods

After Mayo Clinic institutional review board approval, 213 patients with MM diagnosed consecutively between 1/1/2005 and 12/31/2016 receiving early transplant (i.e., frontline ASCT within 1 year of diagnosis) and treated with lenalidomide (with or without dexamethasone) as maintenance therapy were included in this retrospective study. The timing of starting maintenance therapy following ASCT and starting dose of lenalidomide was based on routine current practices. Typically, patients were recommended to start lenalidomide 2–4 months after ASCT at an initial daily dose of 10–15 mg. The mSMART 3.0 criteria were used for risk stratification based on cytogenetics features on interphase FISH [5, 10]. Median follow-up and median duration of maintenance therapy were calculated using the reverse Kaplan–Meier estimator method. Response to therapy was determined using the 2016 IMWG criteria [11]. For deepening of response with maintenance, the best-recorded response after initiation of maintenance therapy was used for comparison with the pre-maintenance therapy response. Choice of treatment regimens at first relapse after maintenance therapy was based on individual provider preference and prevalent data for salvage therapies. Second progression-free survival (PFS2) was calculated from the start of post-maintenance therapy until discontinuation of therapy due to progression, therapy-limiting toxicity, or death. The Kaplan–Meier method was used to estimate the median OS, PFS, and PFS2. All statistical analyses were performed using SPSS (version 19; SPSS Inc., Chicago, IL, USA).

## Results

### Baseline patient characteristics

Between January 1st, 2005 and December 31st, 2016, a total of 213 patients receiving post-ASCT lenalidomide maintenance were included in the analysis. The median follow-up was 5.4 (95% CI: 4.9, 5.9) years from diagnosis and 4.6 (95% CI: 4.2, 5) years from the start of maintenance. The baseline characteristics of these patients at diagnosis are reported in Table 1. The median age for the entire cohort at the initiation of maintenance was 60 years (range: 35–76) and 39% (*n* = 84) were female. Cytogenetics data was available in 202 (95%) patients and 63 (31%) patients were found to have high-risk cytogenetics [i.e., *t*(4;14), *t*(14;16), *t*(14;20), del 17p, gain 1q] based on mSMART 3.0, with the remaining 139 (65%) being standard-risk [10].

**Table 1:** Baseline characteristics of patients at diagnosis.

Parameters	All patients (*N* = 213)
***Demographics***	*n* (%)
Median age at time of maintenance	60 (range: 35–76) years
Gender: Female	84 (39)
***Disease characteristics***	
R-ISS stage	
I	32 (15)
II	111 (52)
III	20 (9)
Not available	50 (23)
***ISS stage***	
I	65 (31)
II	72 (34)
III	55 (26)
Not available	21 (10)
***Cytogenetic risk***	
Standard risk	139 (65)
High risk	63 (30)
• t(4;14)	7 (3)
• t(14;16)	14 (6)
• t(14;20)	5 (2)
• del(17p)	17 (7)
• gain(1q)	29 (12)
Not available	11 (5)
***Therapy prior to ASCT***	
Patients who received IMiDs	149 (70)
Patients who received thalidomide	9 (4)
Patients who received lenalidomide	140 (66)
***Lines of therapies before maintenance***	
1	170 (80)
2	34 (16)
≥ 3	10 (5)
***No. of lines of IMiD-based therapies before maintenance***	
0	64 (30)
1	140 (66)
2	8 (4)
≥ 3	1 (0)
***Median time to transplant from diagnosis (months)***	6.2 (range: 2.8–12)

*ASCT* autologous stem cell transplantation, *IMiD* immunomodulatory drugs, *R-ISS* revised international staging system.

**Fig. 1:**
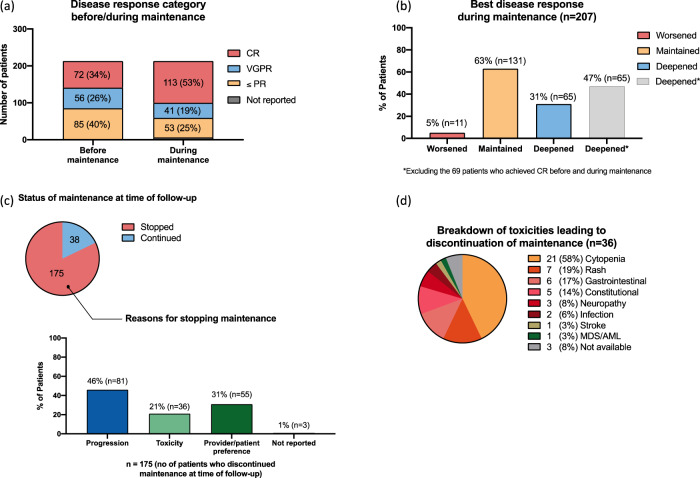
Best response to lenalidomide maintenance and reasons for discontinuing maintenance. **a**, **b** We were unable to calculate the best response during lenalidomide maintenance for 6 (3%) patients. Of the remaining 207 patients, 65 (31%) patients had a deepening of response, 131 (63%) patients maintained their response, and 11 (5%) patients progressed at first reassessment. **c** Lenalidomide was discontinued in 175 (82%) patients. The main reasons for discontinuing lenalidomide were progression (*n* = 81; 46%), provider/patient preference (*n* = 55; 31%), and toxicity (*n* = 36; 21%). The reason for discontinuing lenalidomide maintenance was not reported in 3 (1%) patients. **d** The most common toxicities encountered were cytopenia (*n* = 21; 58%), followed by a rash (*n* = 7; 19%), and gastrointestinal symptoms (*n* = 6; 17%).

One-hundred-forty-nine (70%) patients received immunomodulatory drugs (IMiDs) prior to ASCT. One-hundred-forty (66%) received lenalidomide while 9 (4%) patients received thalidomide. The median time to ASCT from diagnosis was 6.2 (range 2.8–12) months (Table 1). One-hundred-twenty-eight (60%) patients achieved a very good partial response or better (≥VGPR) prior to the initiation of lenalidomide maintenance; with 72 (34%) patients achieving complete response (CR) (Fig. 1a). The median time to starting maintenance therapy was 9.9 (interquartile range: 8.6, 11.6) months from diagnosis and 3.4 (interquartile range: 3.1, 3,9) months from ASCT (Table 2). Ninety-one (43%) patients started maintenance within 100 days of ASCT. One-hundred-ninety (89%) patients received lenalidomide alone while 23 (11%) patients received lenalidomide plus dexamethasone (Rd) as maintenance therapy (Table 2).

**Table 2:** Characteristics of patients at or during maintenance.

Parameters	All patients (*N* = 213)	
Median time to start of maintenance from diagnosis (months)	9.9 (IQR: 8.6, 11.6)	
Median time to start of maintenance from ASCT (months)	3.4 (IQR: 3.1, 3.9)	
***Maintenance regimen***		
Lenalidomide alone	190	89
Lenalidomide + dexamethasone	23	11
***Lenalidomide starting dose***		
5 mg/d	6	3
10 mg/d	96	45
15 mg/d	91	43
20 mg/d	1	0
25 mg/d	14	7
Not available	5	2
Dose reductions	90	44
Duration of maintenance (median years)	1.8 (range: 0.1–10.1)	
Duration < 3 years	165	77
Duration ≥ 3 years	48	23
Patients who discontinued maintenance	175	82
Progression	81	38
Toxicity	39	18
Provider/patient preference	55	26

*ASCT* autologous stem cell transplantation, *IQR* interquartile range.

### Outcome of lenalidomide maintenance post-ASCT

The median duration of maintenance therapy was 1.8 (95% CI: 1.6, 2) years. Response assessment while on lenalidomide maintenance therapy was available for 207 patients (97%); 65 (31%) patients had a deepening of response (i.e., any improvement in IMWG response category), 131 (63%) patients maintained the response achieved after ASCT, and 11 (5%) patients had progression at first reassessment after starting lenalidomide (Fig. 1a and b). At last follow-up, 175 (82%) patients had discontinued maintenance. The main reasons for discontinuing lenalidomide were disease progression (*n* = 81; 46%), provider/patient preference (*n* = 55; 31%), and unacceptable toxicity despite dose modification (*n* = 36; 21%) (Fig. 1c). The reason for discontinuing lenalidomide maintenance was not reported in 3 (1%) patients. At the time of data cutoff, 60 (28%) patients had died and the median OS was not reached. The 5-year OS was 77% from diagnosis and 71% from the start of maintenance (Supplementary Fig. S1a). One-hundred-thirty-six (64%) patients experienced a first relapse and the median PFS was 4 (95% CI: 3.4, 4.5) years from diagnosis and 3 (95% CI: 2.6, 3.5) years from the start of maintenance (Supplementary Figure S1b).

### Factors impacting outcome in patients receiving lenalidomide maintenance

#### High risk cytogenetics and R-ISS stage

Patients with high-risk cytogenetics had a median OS of 8 (95% CI: 4.9, 11) years from diagnosis (5-year OS: 65%) while the median OS for patients with standard-risk cytogenetics was not reached (5-year OS: 82%; *p* = 0.007). Median PFS from diagnosis was 3.3 (95% CI: 2.5, 4.1) years (5-year PFS: 22%) in the high-risk group compared with 4.4 (95% CI: 3.5, 5.4) years (5-year PFS: 44%; *p* = 0.004) in the standard-risk group (Table 3). ISS stage 3 was associated with inferior 5-year OS from diagnosis (62%) compared with ISS stages 1 and 2 (84%; *p* = 0.017). PFS was not significantly different between ISS stage 3 versus ISS stages 1 and 2 (*p* = 0.097). The use of lenalidomide as part of induction therapy prior to ASCT did not significantly impact OS (*p* = 0.88) or PFS (*p* = 0.4) (Table 3).

**Table 3:** Factors impacting outcome in patients receiving lenalidomide maintenance.

Factors impacting outcome		Years from diagnosis				Years from start of maintenance			
		Median OS (95% CI)	*p*	Median PFS (95% CI)	*p*	Median OS(95% CI)	*p*	Median PFS (95% CI)	*p*
**Cytogenetics**	High-risk	8 (4.9–11) (5 y OS: 65%)	**0.007**	3.3 (2.5–4.1) (5 y PFS: 22%)	**0.004**	7.3 (3.9–10.7) (5 y OS: 58%)	**0.009**	2.4 (1.7–3) (5 y PFS: 10%)	**0.006**
	Standard-risk	Not reached (5 y OS: 82%)		4.4 (3.5–5.4) (5 y PFS: 44%)		Not reached (5 y OS: 76%)		3.4 (2.7–4.1) (5 y PFS: 35%)	
**ISS**	Stage 3	Not reached (5 y OS: 62%)	**0.017**	3 (1.6–4.4) (5 y PFS: 28%)	0.097	Not reached (5 y OS: 58%)	**0.022**	2.1 (0.8–3.4) (5 y PFS: 23%)	0.118
	Stage 1 and 2	Not reached (5 y OS: 84%)		4.4 (3.7–5.1) (5 y PFS: 42%)		Not reached (5 y OS: 77%)		3.4 (2.8–4) (5 y PFS: 31%)	
**Lenalidomide at induction**	Yes	Not reached (5 y OS: 77%)	0.884	3.7 (2.7–4.7) (5 y PFS: 42%)	0.409	8.1 (NR, NR) (5 y OS: 73%)	0.946	3.2 (2.5–3.9) (5 y PFS: 32%)	0.436
	No	Not reached (5 y OS: 76%)		4 (3.2–4.7) (5 y PFS: 28%)		Not reached (5 y OS: 66%)		2.9 (1.9–4) (5 y PFS: 21%)	
**Deep response within 2 years of starting maintenance**	≥VGPR	Not reached (5 y OS: 82%)	**0.003**	4.4 (3.9–4.9) (5 y PFS: 41%)	**0.003**	Not reached (5 y OS: 76%)	**0.003**	3.6 (3.1–4.1) (5 y PFS: 32%)	**0.003**
	≤PR	8 (5.5–10.4) (5 y OS: 67%)		3.3 (2.2–4.3) (5 y PFS: 30%)		8.3 (4.7–9.9) (5 y OS: 59%)		2.6 (1.6–3.6) (5 y PFS: 20%)	
**Year of maintenance initiation**	≥2014	Not reached (5 y OS: 89%)	**<0.0001**	4.3 (3.9–4.8) (5 y PFS: 40%)	0.59	Not reached (5 y OS: 78%)	**<0.0001**	3.4 (2.8–4) (5 y PFS: 23%)	0.58
	<2014	8.2 (not estimable) (5 y OS: 64%)		3.5 (2.8–4.2) (5 y PFS: 35%)		7.5 (not estimable) (5 y OS: 61%)		2.6 (1.9–3.3) (5 y PFS: 28%)	
**Timing of first achievement of** **≥** **VGPR**	Prior to mx	Not reached (5 y OS: 77%)	0.405	4.3 (3.5–5.2) (5 y PFS: 33%)	0.166	Not reached (5 y OS: 71%)	0.401	3.1 (2.2–4) (5 y PFS: 26%)	0.152
	Within 2 years of mx	Not reached (5 y OS: 80%)		5 (3.6–6.5) (5 y PFS: 54%)		Not reached (5 y OS: 75%)		4.4 (2.7–6) (5 y PFS: 40%)	

*CI* confidence interval, *ISS* international staging system, *Mx* maintenance, *OS* overall survival, *PFS* progression free survival, *VGPR* very good partial response.

#### Impact of deeper response (i.e., ≥VGPR) during maintenance on PFS and OS

One-hundred-twenty-eight (60%) patients achieved ≥ VGPR post-ASCT prior to starting of maintenance (Fig. 1a). Of these, 82 (64%) received lenalidomide during induction. Eighty-five (40%) patients achieved ≤ PR post-ASCT prior to maintenance and of these, 58 (68%) patients received lenalidomide induction (*p* = 0.53).

Response assessment while on lenalidomide maintenance therapy was not available for 6 patients (3%). Of the remaining 207 patients, 154 (74%) patients achieved or maintained ≥VGPR as the best response while on lenalidomide maintenance (Fig. 1a). One-hundred-fifty-two (73%) patients achieved or maintained a best response of ≥VGPR within 2 years of starting maintenance and 2 (1%) patients achieved ≥ VGPR more than 2 years after the start of maintenance.

Patients who achieved or maintained a best response of ≥VGPR within 2 years of maintenance were noted to have a significantly better OS from diagnosis (5-year OS: 82%) compared with patients with ≤PR (67%; *p* = 0.003) (Table 3). The median PFS from diagnosis was 4.4 (95% CI: 3.9, 4.9) years in patients with ≥ VGPR as a best response within 2 years of maintenance versus 3.3 (95% CI: 2.2, 4.3) years in patients with ≤ PR (*p* = 0. 003) (Table 3). Adjusting for age, ISS stage 3, cytogenetic risk group, and patients who received Rd maintenance, the hazard ratio (HR) for OS and PFS in patients who achieved or maintained a best response of ≥ VGPR within 2 years of maintenance was 0.32 (95% CI: 0.18, 0.58; *p* < 0.0001) and 0.56 (95% CI: 0.38, 0.83; *p* = 0.004), respectively.

There was no significant difference in OS and PFS between patients who achieved ≥ VGPR prior to the start of maintenance (*n* = 128) compared with patients who first achieved ≥ VGPR (i.e., had deepening of response to ≥VGPR) within the first 2 years of lenalidomide maintenance (*n* = 35). The 5-year OS from diagnosis was 77% in patients who achieved ≥VGPR prior to maintenance compared with 80% in patients who first achieved ≥ VGPR within the first 2 years of maintenance (*p* = 0.41). Median PFS from diagnosis was 4.3 (95% CI: 3.5, 5.2) years in patients who achieved ≥ VGPR prior to maintenance versus 5 (95% CI: 3.6, 6.5) years (*p* = 0.17) in patients who first achieved ≥ VGPR within the first 2 years of maintenance (Table 3).

### Impact of duration of maintenance on survival

**Fig. 2:**
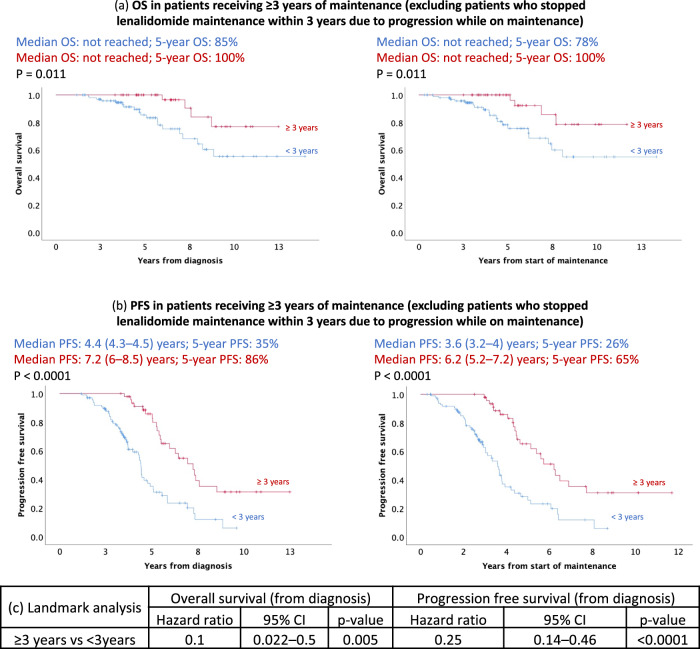
Progression free survival (PFS) and overall survival (OS) in patients receiving ≥ 3 years of lenalidomide maintenance (excluding patients who stopped lenalidomide maintenance within 3 years due to progression while on maintenance). **a** The 5-year OS from diagnosis in patients who received ≥3 years of maintenance was 100% versus 85% in patients who received <3 years of maintenance (*p* = 0.011). 5-year OS from the start of maintenance was 100% in the ≥3 year cohort versus 78% the <3 year group (*p* = 0.011). **b** Median PFS in patients who received ≥ 3 years of maintenance was 7.2 (95% CI: 6, 8.5) years (5-year PFS: 86%) from diagnosis versus 4.4 (95% CI: 4.3, 4.5) years (5-year PFS: 35%) in patients who received <3 years of maintenance (*p* < 0.0001). Median PFS from the start of maintenance was 6.2 (95% CI: 5.2, 7.2) years (5-year PFS: 65%) in patients who received lenalidomide for >3 years versus 3.6 (95% CI: 3.2, 4) years (5-year PFS: 26%) in those who received <3 years of maintenance (*p* < 0.0001). **c** Adjusting for age, ISS stage 3, cytogenetic risk, and patients who received Rd maintenance, HR for OS was 0.1 (95% CI: 0.022, 0.5) in favor of maintenance ≥3 years (*p* = 0.005) and the HR for PFS was 0.25 (95% CI: 0.14, 0.46; *p* < 0.0001) in favor of maintenance ≥3 years.

We performed landmark analysis at 3 years post-initiation of maintenance. Sixty-five (31%) patients who stopped lenalidomide maintenance within 3 years due to progression on maintenance were excluded from the analysis to control for guaranteed-time bias. Excluding these patients, those who received ≥3 years of maintenance (*n* = 48) had superior 5-year OS from diagnosis of 100% versus 85% in patients who received <3 years of maintenance (*n* = 100) (*p* = 0.011) (Fig. 2a). Median PFS from diagnosis was 7.2 (95% CI: 6, 8.5) years (5-year PFS: 86%) in patients who received lenalidomide for more than 3 years vs. 4.4 (95% CI: 4.3, 4.5) years (5-year PFS: 35%; *p* < 0.0001) in those who received <3 years of maintenance (Fig. 2b). The proportion of high-risk cytogenetics was 21% in ≥3 years cohort and 23% in the <3 years cohort (*p* = 0.82). The proportion of ISS stage III was 27% in the ≥3 years cohort vs. 18% in the <3 years group (*p* = 0.19). 15% of patients in the ≥3 years cohort received Rd compared with 9% in the <3 years group (*p* = 0.31) (Supplementary Table S1). Adjusting for age, ISS stage 3, cytogenetic risk, and patients who received Rd maintenance, HR for OS and PFS were 0.1 (95% CI: 0.022, 0.5; *p* = 0.005) and 0.25 (95% CI: 0.14, 0.46; *p* < 0.0001), respectively, in favor of maintenance ≥3 years (Fig. 2c).

Median OS was significantly better in patients who started lenalidomide on or after 01/01/2014 (not reached; 5 y OS: 89%; *n* = 124) compared to those who started lenalidomide maintenance on or before 12/31/2013 [8.2 (not estimable) months; 5 y OS: 64%; *n* = 89; *p* < 0.0001) (Table 3). Median PFS was not significantly different between the two groups (*p* = 0.59).

### Adverse effects during maintenance therapy

Lenalidomide was dose reduced in 89 (43%) patients during maintenance and data for dose modification was not available in 8 patients. The most common adverse effects that resulted in discontinuation of lenalidomide maintenance were cytopenia (*n* = 21; 58%), followed by a rash (*n* = 7; 19%), and gastrointestinal symptoms (*n* = 6; 17%) (Fig. 1d). A total of 8 (4%) patients developed second primary malignancies (SPMs) (excluding non-melanomatous cutaneous malignancies) after the start of lenalidomide maintenance. Specifically, 4 patients developed hematologic malignancies and 4 patients developed solid SPMs (Supplementary Table S2).

### Outcomes with salvage regimens at first relapse post-maintenance

One-hundred-thirty-six (64%) patients relapsed during or after stopping maintenance; 126 (59%) patients were documented to have received therapy at relapse (10 patients were either lost to follow-up or opted for no treatment).

#### Impact of lenalidomide refractoriness at first relapse

**Fig. 3:**
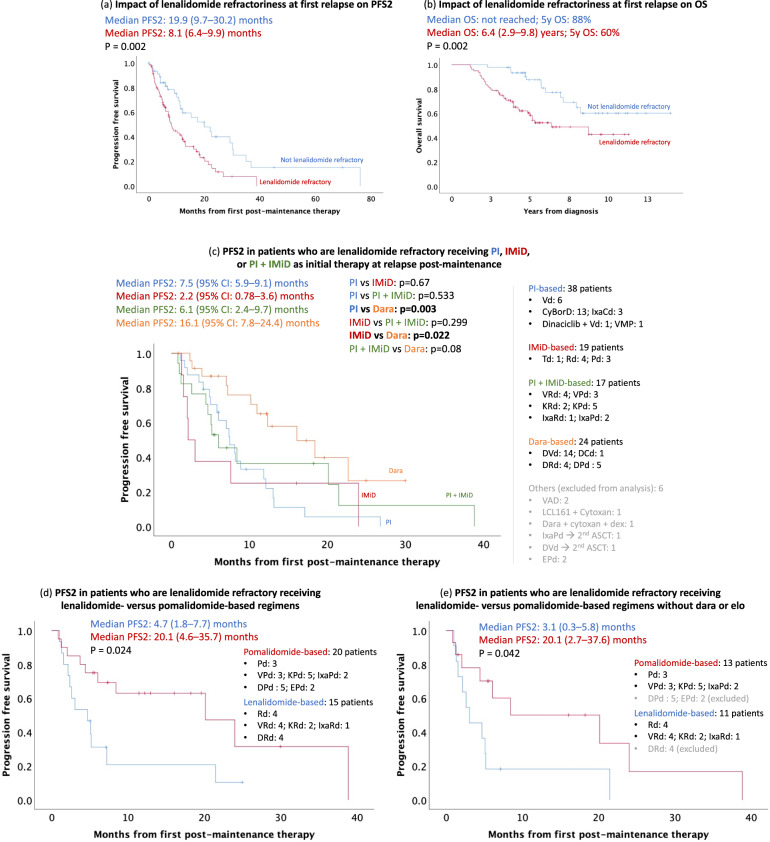
Impact of lenalidomide resistance at time of salvage on overall survival (OS) and progression free survival (PFS). **a** The median PFS2 was 8.1 (95% CI: 6.4, 9.9) months in patients who were lenalidomide refractory compared to 19.9 (95% CI: 9.7, 30.2) months in those who were not (*p* = 0.002). **b** The median overall survival (OS) in lenalidomide refractory was 6.4 (95% CI: 2.9, 9.8) years (5-year OS: 60%) in patients who were lenalidomide refractory but was not reached (5-year OS: 88%) in patients who were not (*p* = 0.002). Median OS from the start of maintenance was 5.2 (95% CI: 2.4, 8) years (5-year OS: 52%) in patients who were lenalidomide refractory vs not reached (5-year OS: 78%) in patients who were not (*p* = 0.002) (data not shown). **c** In lenalidomide refractory patients, IMiD-based therapies were associated with a median PFS2 of 2.2 (95% CI: 0.78, 3.6) months which was not significantly different from PI-based therapies [7.5 (95% CI: 5.9, 9.1) months; *p* = 0.67] or PI + IMiD-based therapies [6.1 (95% CI: 2.4, 9.7) months; *p* = 0.299). Dara- based therapies were associated with a median PFS2 of 16.1 (95% CI: 7.8, 24.4) months which was significantly superior to IMiD-based (*p* = 0.022) and PI-based (*p* = 0.003) therapies but not PI + IMiD-based therapies (*p* = 0.08). **d** In lenalidomide refractory patients, pomalidomide-based therapies were associated with superior PFS2 compared to lenalidomide-based therapies [20.1 (95% CI: 4.6, 35.7) months versus 4.7 (95% CI: 1.8, 7.7) months; *p* = 0.024]. **e** Excluding patients receiving daratumumab or elotuzumab, median PFS2 of pomalidomide-based regimens [20.1 (95% CI: 2.7, 37.6) months] was superior to lenalidomide-based therapies [3.1 (95% CI: 0.3, 5.8) months; *p* = 0.042].

Of the 126 patients who received salvage therapy, 80 (63%) patients relapsed while on maintenance or progressed within 60 days of stopping lenalidomide maintenance (i.e., lenalidomide refractory) and 46 (37%) patients stopped maintenance >60 days before the next therapy (i.e., not lenalidomide refractory). A comparison of the therapies used at first relapse post-maintenance between these two groups can be found in Supplementary Table S3. A total of 25% (*n* = 20; response could not be determined for 1 patient) of lenalidomide refractory patients achieved ≥VGPR to salvage therapy at first relapse post-maintenance compared to 39% (*n* = 17; response could not be determined for 2 patients) for patients that were not lenalidomide refractory at relapse (*p* = 0.12). The median PFS2 was 8.1 (95% CI: 6.4, 9.9) months in patients who were lenalidomide refractory compared to 19.9 (95% CI: 9.7, 30.2; *p* = 0.002) months in those who were not (Fig. 3a). Adjusting for age, ISS stage 3, cytogenetic risk, and patients who received Rd maintenance, lenalidomide refractoriness at relapse was associated with inferior PFS2 [HR 1.9 (95% CI: 1.1, 3.2; *p* = 0.017)]. The 5-year OS from diagnosis was 60% in patients who were lenalidomide refractory compared with 88% in patients who were not (*p* = 0.002) and HR for OS was 2.4 (95% CI: 1.2, 5 months; *p* = 0.019) (Fig. 3b). In patients who were refractory to lenalidomide at first relapse and did not receive daratumumab, there was no significant difference in median PFS2 between IMiD-based salvage therapies [2.2 (95% CI: 0.78, 3.6) months; *n* = 19] versus proteasome inhibitor (PI)-based salvage therapies [7.5 (95% CI: 5.9, 9.1) months; *n* = 38; *p* = 0.67] or PI + IMiD-based therapies [6.1 (95% CI: 2.4, 9.7; *n* = 17) months; *p* = 0.3] (Fig. 3c). The use of pomalidomide-based regimens including daratumumab or elotuzumab in lenalidomide refractory patients was associated with superior median PFS2 compared to lenalidomide-based therapies including daratumumab or elotuzumab [20.1 (95% CI: 4.6, 35.7) months vs. 4.7 (95% CI: 1.8, 7.7) months; *p* = 0.024] (Fig. 3d). Consistent with this, response to lenalidomide-based salvage therapies was poorer in patients who were lenalidomide refractory [median PFS2: 0.6 (95% CI: 0.3, 0.9) years] compared to nonrefractory patients [median PFS2: 1.8 (95% CI: 0.8, 2.8) years; *p* = 0.006]. Excluding patients who received daratumumab or elotuzumab, median PFS2 of pomalidomide-based regimens [20.1 (95% CI: 2.7, 37.6) months] was still superior to lenalidomide-based therapies [3.1 (95% CI: 0.3, 5.8) months; *p* = 0.042] in lenalidomide refractory patients (Fig. 3e).

#### Daratumumab vs non-daratumumab regimens

**Fig. 4:**
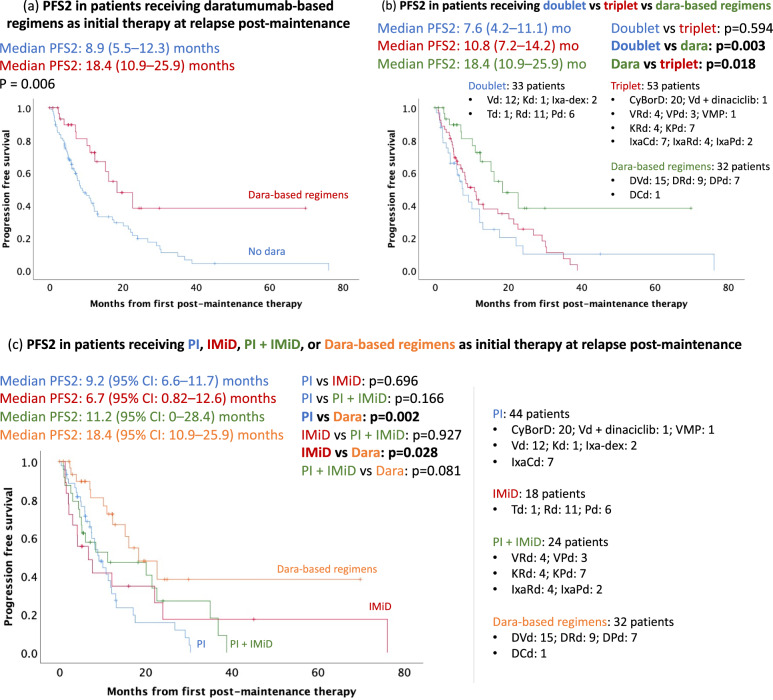
Daratumumab-based regimens vs. non-daratumumab-based combinations at first relapse post-maintenance. **a** Daratumumab-based regimens outperformed combinations without daratumumab. The median PFS2 was significantly longer in patients who received daratumumab-based regimens [18.4 (95% CI: 10.9, 25.9) months] compared to patients who did not receive daratumumab [8.9 (95% CI: 5.5, 12.3) months; *p* = 0.006]. **b** Without daratumumab, doublet therapy is comparable to triplet therapy. Median PFS2 in patients who received doublet was 7.6 (95% CI: 4.2, 11.1) months versus 10.8 (95% CI: 7.2, 14.2) months in patients who received triplet (*p* = 0.59). The use of daratumumab-based regimens was associated with a superior median PFS2 of 18.4 (95% CI: 10.9, 25.9) months (*p* < 0.003 compared to doublet; *p* = 0.018 compared to triplet). **c** In patients not receiving daratumumab, there was no significant difference in median PFS2 between those who received PI-based combinations [9.2 (95% CI: 6.6, 11.7) months; *n* = 44] compared to IMiD-based combinations [6.7 (95% CI: 0.82, 12.6) months; *n* = 18; *p* = 0.7] or PI + IMiD-based combinations [11.2 (95% CI: 0, 28.4 months; *n* = 24; *p* = 0.17].

Of the 126 patients who received salvage therapy, 8 patients were excluded from subsequent analyses to ensure uniformity of the treatment groups (Supplementary Table S4). Of the remaining 118 patients, 32 (27%) patients received daratumumab-based regimens at first relapse and 86 (73%) patients did not receive daratumumab. The median PFS2 was significantly longer in patients who received daratumumab-based regimens [18.4 (95% CI: 10.9, 25.9) months] compared to patients who did not receive daratumumab [8.9 (95% CI: 5.5, 12.3) months; *p* = 0.006] (Fig. 4a). Specifically, daratumumab-based regimens were associated with superior median PFS2 when compared to both doublet (*n* = 33) [7.6 (95% CI: 4.2, 11.1) months; *p* = 0.003] and triplet (*n* = 53) [10.8 (95% CI: 7.2, 14.2) months; *p* = 0.018] combinations without daratumumab (Fig. 4b). Adjusting for age, ISS stage 3, cytogenetic risk, patients who received Rd maintenance, year of initiation of lenalidomide maintenance (before or after 1/1/2014), and lenalidomide refractoriness at salvage, daratumumab-based regimens were associated with improved median PFS2 [HR 0.35 (95% CI: 0.17, 0.68; *p* = 0.002)]. In patients who did not receive daratumumab (*n* = 86), 33 (38%) received doublet therapy and 53 (62%) received triplet therapy. There was no significant difference in median PFS2 with doublet [7.6 (95% CI: 4.2, 11.1) months] vs. triplet therapy [10.8 (95% CI: 7.2, 14.2); *p* = 0.59] when daratumumab was not part of the treatment regimen at relapse (Fig. 4b). There was also no significant difference in median PFS2 between those who received PI-based combinations [9.2 (95% CI: 6.6, 11.7) months; *n* = 44] compared to IMiD-based combinations [6.7 (95% CI: 0.82, 12.6) months; *n* = 18; *p* = 0.7] or PI + IMiD-based combinations [11.2 (95% CI: 0, 28.4 months; *n* = 24; *p* = 0.17] without daratumumab (Fig. 4c). In non-daratumumab-based regimens, there was no significant difference in median PFS2 between patients who received lenalidomide-based combinations [6.7 (95% CI: 0, 15.4) months; *n* = 23] compared with pomalidomide-based regimens [20.1 (95% CI: 0, 41.4) months; *n* = 18; *p* = 0.5].

#### Daratumumab + IMiD vs daratumumab + PI (daratumumab + bortezomib + dexamethasone)

**Fig. 5:**
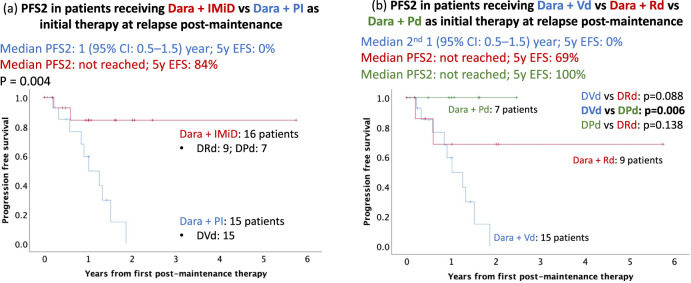
Daratumumab + IMiD vs daratumumab + PI at first relapse post-maintenance. Daratumumab + IMiD compared with daratumumab + PI at first relapse after maintenance (**a**) The median PFS2 in patients who received daratumumab + IMiD (*n* = 16) was not reached compared with 1 (95% CI: 0.5, 1.5) year in patients who received daratumumab + PI (*n* = 15) (*p* = 0.004). There were 84% of patients event-free at 5 years in the dara + IMiD group and 0% in the dara + PI group. **b** The median PFS2 in patients who received dara + Rd or dara + Pd were not reached. 5-year event-free survival was 69% in the dara + Rd group versus 100% in dara + Pd (*p* = 0.138). Dara + Pd was associated with a significantly better 5-year EFS (100%) compared with dara + Vd (0%; *p* = 0.006).

The median PFS2 in patients who received daratumumab + IMiD (*n* = 16) was not reached compared with 1 year (95% CI: 0.5, 1.5 years) in patients who received daratumumab + PI (*n* = 15) (*p* = 0.004) (Fig. 5a). In the small subset of patients with daratumumab + IMiD combination, there was no significant difference in 5-year event-free survival (EFS) in patients who received daratumumab + Rd (DRd) (5-year EFS: 69%; *n* = 9) compared to daratumumab + pomalidomide + dexamethasone (DPd) (5-year EFS: 100%; *n* = 7; *p* = 0.14) (Fig. 5b). Patients receiving DPd had a longer median PFS2 (not reached) compared to daratumumab + bortezomib + dexamethasone (DVd) [1 (95% CI: 0.5, 1.5) year; *p* = 0.006] (Fig. 5b). The DRd group also had a longer median PFS2 (not reached) compared to DVd, but this was not statistically significant (*p* = 0.088) (Fig. 5b). The clinical characteristics, including the proportion of high-risk (27% versus 31%; *p* = 0.78) and ISS stage III at diagnosis (20% versus 38%; *p* = 0.4) were comparable between daratumumab + PI vs. daratumumab + IMiD therapy, respectively (Supplementary Table S5). The proportion of patients who were lenalidomide refractory at salvage was significantly higher in the daratumumab + PI (93%) cohort compared to the daratumumab + IMiD cohort (56%; *p* = 0.018). Adjusting for age, ISS stage 3, cytogenetic risk, patients who received Rd maintenance, and lenalidomide refractoriness at time of salvage, the daratumumab + IMiD had a superior PFS2 compared to daratumumab + PI [HR 0.1 (95% CI: 0.016, 0.58); *p* = 0.011].

## Discussion

Lenalidomide maintenance is an effective and well-tolerated strategy to prolong PFS even in the era of novel agents alongside ASCT, and several trials have demonstrated a remarkable risk reduction in progression and death especially in the post-ASCT setting [1, 3, 4, 6]. However, several important questions on the ideal duration of maintenance, as well as the optimal choice of therapy at first relapse remain unanswered.

The PFS and OS data in our uniform cohort was largely comparable to the previously reported outcomes in phase 3 trials studying lenalidomide maintenance. The median PFS in our study was 3 years (i.e., 36 months) from the start of maintenance and 4 years (i.e., 48 months) from diagnosis, which is comparable to the median PFS reported in the Myeloma IX (39 months from randomization), IFM (41 months from randomization), CALGB (46 months from randomization), and GEMIMA (42 months from randomization) studies [1, 3, 4, 6]. The 3 year OS from the start of maintenance was 84% (89% from diagnosis) which is comparable to the 3 year OS reported in Myeloma IX (79%), IFM (80%), CALGB (88%), and GEMIMA (88%) [1, 3, 4, 6]. In our cohort, achievement or maintenance of a deeper response (≥VGPR) within 2 years of maintenance therapy was associated with an improvement in PFS and OS. This is consistent with multiple prior studies demonstrating improvement in outcomes with deeper responses [12–14].

Our study also suggests that a longer duration of lenalidomide maintenance (i.e., ≥3 years) was associated with an improved OS and PFS, and this association was significant even after adjusting for high-risk cytogenetics, age, and ISS stage 3. We controlled for guarantee-time bias by excluding patients that had stopped lenalidomide maintenance within 3 years due to disease progression. Consistent with our results, a pooled analysis of the GIMEMA MM-03–05, RV-MM-PI-209, and CC-5013-MM-015 trials showed that continuous therapy (defined as upfront therapy followed by maintenance lasting ≥ 2 years) was superior to fixed duration therapy (defined as upfront treatment for ≤1 year) [8].

Patients who started maintenance therapy on or after 1/1/2014 (*n* = 124) had significantly better OS compared with patients who started maintenance on or before 12/31/2013 (*n* = 89; *p* < 0.0001), which is most probably explained by the availability and use of novel therapies. We next compared the efficacy of various groups of therapies in the post-maintenance relapse setting and found that, consistent with the above, the use of daratumumab (first FDA-approved in 2015) at initial relapse was associated with improved PFS2. Daratumumab in combination with an IMiD was significantly superior compared to daratumumab with bortezomib, even after adjusting for age, high-risk cytogenetics, ISS stage 3, and lenalidomide refractoriness at time of salvage. One possible explanation for the synergism between daratumumab and IMiDs is the latter’s ability to enhance NK cell proliferation, cytotoxic activity, and therefore daratumumab-mediated antibody-dependent cellular cytotoxicity (ADCC); even in the setting of IMiD-refractory MM cells [15, 16]. Consistent with this, prior preclinical and clinical studies show that the addition of IMiDs to daratumumab can overcome daratumumab refractoriness in lenalidomide refractory patients [17, 18]. Specifically, 33% of daratumumab and pomalidomide double-refractory patients responded to DPd in the clinical study [17].

In a relatively small subset of patients treated with daratumumab in combination with an IMiD (*n* = 16), the choice of IMiD [lenalidomide (*n* = 9) vs pomalidomide (*n* = 7)] did not impact PFS2 but the strength of this finding is limited by the small numbers. We also found that DPd (*n* = 7) was associated with a significantly improved median PFS2 compared to DVd (*n* = 15) (*p* = 0.006) and that DRd (*n* = 9) had a longer, but statistically insignificant, median PFS2 compared to DVd (*p* = 0.088). Again, the strength of these findings is limited by the small numbers. When daratumumab was not utilized at first relapse, the choice of regimens (doublet vs. triplet, PI vs IMiDs, lenalidomide vs pomalidomide) did not impact the PFS2. This data is especially important to guide treatment choice in resource-limited settings where cost is often a limiting factor for using daratumumab. Thirty-nine (18%) patients in our study discontinued maintenance due to adverse events, which was lower than the IFM and myeloma IX studies (27% and 28%, respectively) but higher than the CALGB and GEMIMA studies (10% and 5.2%, respectively) [2–4].

Our study is limited by its retrospective nature and the inherent biases this introduces in the interpretation of these results, especially for toxicity and response assessment data. While largely uniform including only those patients with early ASCT and lenalidomide-based maintenance, there is a small proportion of patients who received lenalidomide + dexamethasone (*n* = 23; 11%) as the maintenance regimen. Additionally, the current changing patterns of maintenance therapy for high-risk diseases (e.g., triplet maintenance, consolidation, and tandem ASCT) may limit the applicability of our data to this study population. The impact of newer therapies, including immunotherapies and bispecific antibodies, on long-term outcomes with lenalidomide maintenance is difficult to assess from our data given the limited number of patients exposed to these newer therapies. Nonetheless, our study does provide valuable comparative information in choosing appropriate agents at relapse on maintenance therapy.

## Conclusion

Maintenance therapy with lenalidomide is well tolerated with a longer duration (≥3 years) being associated with improvement in PFS and OS. Daratumumab-based therapies at relapse have a significant improvement in PFS2, with the daratumumab-IMiD combination demonstrating improved PFS2 compared to the daratumumab-bortezomib combination.

## Supplementary information

Supplementary Document
