# Supplementary material for: The Effect of Duration of Lenalidomide Maintenance and Outcomes of Different Salvage Regimens in Patients with Multiple Myeloma (MM)
Source: Blood Cancer J. 2021 Sep 22;11(9):158. doi: 10.1038/s41408-021-00548-7 (PMC8458275; doi:10.1038/s41408-021-00548-7)
Supplement: Supplementary file 1 — Supplementary Document [file 41408_2021_548_MOESM1_ESM.pdf]

(a) **OS in patients receiving ≥ 3 months of lenalidomide maintenance**

Median OS: Not reached; 5y OS: 77%

Median OS: not reached; 5y OS: 71%

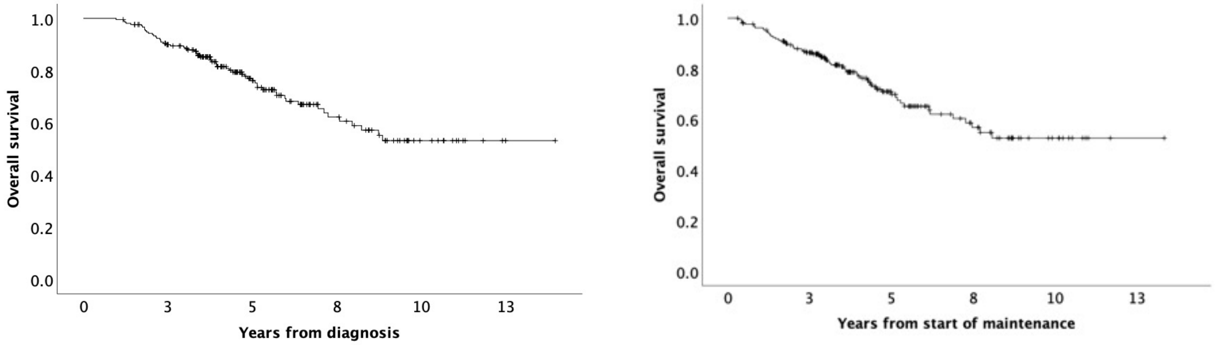

(b) **PFS in patients receiving ≥ 3 months of lenalidomide maintenance**

Median PFS: 4 (3.4–4.5) years; 5y PFS: 38%

Median PFS: 3 (2.6–3.5) years; 5y PFS: 28%

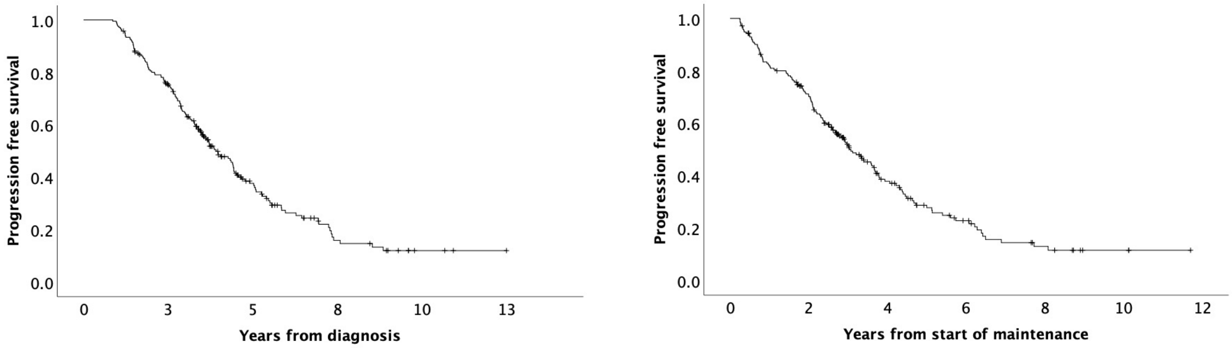

Supplementary Figure S1: OS and PFS in patients receiving ≥ 3 months of lenalidomide maintenance
(a) The median OS in all patients receiving at least 3 months of lenalidomide maintenance was 14.1 (95% CI: NR, NR) from diagnosis. Median OS from start of maintenance was not reached. 5-year OS was 77% from diagnosis and 71% from start of maintenance. (b) The median PFS in patients receiving at least 3 months of lenalidomide maintenance was 4 (95% CI: 3.4, 4.5) years (5-year PFS: 38%) from diagnosis and 3 (95% CI: 2.6, 3.5) years (5-year PFS: 28%) from start of maintenance.

Supplementary Table S1: Subgroup analysis of clinical characteristics at diagnosis stratified by duration of lenalidomide maintenance

		Excluding patients who stopped maintenance within 3 years due to progression					
		**All durations**		**< 3 years**		**≥ 3 years**	
		n=148	%	n=100	%	n=48	%
**Gender, female**		59	40	42	42	17	35
**Median age, years (range)**		60 (35–76) years		60 (35–76) years		61 (43–74) years	
**R-ISS**	**I**	26	18	17	17	9	19
	**II**	77	52	56	56	21	44
	**III**	7	5	4	4	3	6
	**Not available**	38	26	23	23	15	31
**ISS**	**I**	52	35	35	35	17	35
	**II**	50	34	37	37	13	27
	**III**	31	21	18	18	13	27
	**Not available**	15	10	10	10	5	10
**Cytogenetics**	**Standard-risk**	105	71	71	71	34	71
	**High-risk**	33	22	23	23	10	21
	**Not available**	10	7	6	6	4	8
**Maintenance regimen**	**Len alone**	132	89	91	91	41	85
	**Len + dex**	16	11	9	9	7	15

Supplementary Table S2: Second primary malignancies (SPMs) during or after lenalidomide maintenance

SPM	n=8	%	Duration of maintenance (months)*
Solid malignancy	***4***	***1.9***	
Breast cancer	2	0.9	8.3
			21.8
Colon cancer	1	0.5	35.2
Cholangiocarcinoma	1	0.5	55.0
Hematological malignancy	***4***	***1.9***	
Mycosis fungiodes	1	0.5	5.8
MDS/AML	3	1.4	68.1
			19.3
			53.0
Median, months (range)			28.5 (8.3–68.1)

*Duration of maintenance at the time of SPM diagnosis or total duration of maintenance (for patients who developed SPM after stopping maintenance for other reasons)

Supplementary Table S3: Therapies used at first relapse post maintenance in patients who were lenalidomide refractory compared to patients who were not

	Lenalidomide refractory at first-relapse post-maintenance?			
	Yes		No	
**Regimen used at first relapse post-maintenance**	**n=80**	**%**	**n=46**	**%**
**Proteasome inhibitor-based**	***24***	***30***	***20***	***43***
**Doublet**	***6***	***8***	***9***	***20***
Bortezomib + dexamethasone (Vd)	6	8	6	13
Carfilzomib + dexamethasone (Kd)			1	2
Ixazomib + dexamethasone (Ixad)			2	4
**Triplet**	***18***	***23***	***11***	***24***
Bortezomib + cyclophosphamide + dexamethasone (VCd)	13	16	7	15
Bortezomib + dinaciclib + dexamethasone	1	1		
Bortezomib + melphalan + prednisone (VMP)	1	1		
Ixazomib + cyclophosphamide + dexamethasone (IxaCd)	3	4	4	9
**IMiD-based**	***8***	***10***	***10***	***22***
Thalidomide + dexamethasone (Td)	1	1		
Lenalidomide + dexamethasone (Rd)	4	5	7	15
Pomalidomide + dexamethasone (Pd)	3	4	3	7
**PI + IMiD-based**	***17***	***21***	***7***	***15***
Bortezomib + lenalidomide + dexamethasone (VRd)	4	5		
Bortezomib + pomalidomide + dexamethasone (VPd)	3	4		
Carfilzomib + lenalidomide + dexamethasone (KRd)	2	3	2	4
Carfilzomib + pomalidomide + dexamethasone (KPd)	5	6	2	4
Ixazomib + lenalidomide + dexamethasone (IxaRd)	1	1	3	7
Ixazomib + pomalidomide + dexamethasone (IxaPd)	2	3		
**Monoclonal antibody-based**	***26***	***33***	***8***	***17***
Daratumumab + bortezomib + dexamethasone (DVd)	14	18	1	2
Daratumumab + lenalidomide + dexamethasone (DRd)	4	5	5	11
Daratumumab + pomalidomide + dexamethasone (DPd)	5	6	2	4
Daratumumab + cyclophosphomide + dexamethasone (DCd)	1	1		
Elotuzumab + pomalidomide + dexamethasone (EPd)	2	3		
**Other**	***5***	***6***	***1***	***2***
LCL161 + cyclophosphamide	1	1		
2nd ASCT			1	2
DVd followed by 2nd ASCT	1	1		
IxaPd followed by 2nd ASCT	1	1		
Vincristine, doxorubicin, dexamethasone (VAD)	2	3		

Supplementary Table S4: Regimens used at first relapse post-maintenance

Regimen used at first relapse post-maintenance	n=126	%
**Proteasome inhibitor-based**	***44***	***35***
**Doublet**	***15***	***12***
Bortezomib + dexamethasone	12	10
Carfilzomib + dexamethasone	1	1
Ixazomib + dexamethasone	2	2
**Triplet**	***29***	***23***
Bortezomib + cyclophosphamide + dexamethasone	20	16
Bortezomib + dinaciclib + dexamethasone	1	1
Bortezomib + melphalan + prednisone	1	1
Ixazomib + cyclophosphamide + dexamethasone	7	6
**IMiD-based**	***18***	***14***
Thalidomide + dexamethasone	1	1
Lenalidomide + dexamethasone	11	9
Pomalidomide + dexamethasone	6	5
**PI + IMiD-based**	***24***	***19***
Bortezomib + lenalidomide + dexamethasone	4	3
Bortezomib + pomalidomide + dexamethasone	3	2
Carfilzomib + lenalidomide + dexamethasone	4	3
Carfilzomib + pomalidomide + dexamethasone	7	6
Ixazomib + lenalidomide + dexamethasone	4	3
Ixazomib + pomalidomide + dexamethasone	2	2
**Daratumumab-based**	***32***	***25***
Daratumumab + bortezomib + dexamethasone	15	12
Daratumumab + lenalidomide + dexamethasone	9	7
Daratumumab + pomalidomide + dexamethasone	7	6
Daratumumab + cyclophosphomide + dexamethasone	1	1
**Others (excluded from analyses)**	***8***	***6***
2nd ASCT	1	1
LCL161 + cyclophosphamide	1	1
Elotuzumab + pomalidomide + dexamethasone	2	2
DVd followed by 2nd ASCT	1	1
IxaPD followed by 2nd ASCT	1	1
Vincristine, doxorubicin, dexamethasone	2	2

Supplementary Table S5: Subgroup analysis of clinical characteristics at diagnosis stratified by types of daratumumab combinations

		All daratumumab		Dara + PI		Dara + IMiD		Dara + Cytoxan	
		n=32	%	n=15	%	n=16	%	n=1	%
**Gender, female**		5	16	3	20	2	13	0	0
**Median age, years (range)**		61 (48–74)		60 (56–74)		63 (56–74)		48	
**R-ISS**	**I**	4	13	3	20	1	6		
	**II**	20	63	9	60	10	63		
	**III**	1	3			1	6	1	100
	**Not available**	7	22	3	20	4	25		
**ISS**	**I**	11	34	6	40	5	31		
	**II**	9	28	4	27	5	31	1	100
	**III**	10	31	3	20	6	38		
	**Not available**	2	6	2	13				
**Cytogenetics**	**Standard-risk**	23	72	11	73	11	69	1	100
	**High-risk**	9	28	4	27	5	31		
**Maintenance regimen**	**Len alone**	28	88	13	87	15	94		
	**Len + dex**	4	13	2	13	1	6	1	100
**Len refractory at salvage**	**Yes**	24	75	14	93	9	56	1	100
	**No**	8	25	1	7	7	44		
